# Integrating bulk and single-cell sequencing data to construct a Scissor^+^ dendritic cells prognostic model for predicting prognosis and immune responses in ESCC

**DOI:** 10.1007/s00262-024-03683-9

**Published:** 2024-04-15

**Authors:** Maosheng Cheng, Jianqi Xiong, Qianwen Liu, Caihua Zhang, Kang Li, Xinyuan Wang, Shuang Chen

**Affiliations:** 1 Department of Medical Oncology; Institute of Precision Medicine; Center for Translational Medicine, The First Affiliated Hospital of Sun Yat-Sen University, Guangzhou, 510080 China; 2 State Key Laboratory of Oncology in South China, Department of Thoracic Surgery, Collaborative Innovation Center for Cancer Medicine, Guangdong Esophageal Cancer Institute, Sun Yat-Sen University Cancer Center, Guangzhou, China; 3 The Third Affiliated Hospital, Sun Yat-Sen University, Guangzhou, China

**Keywords:** ESCC, DC, SCISSOR

## Abstract

Esophageal squamous cell carcinoma (ESCC) is characterized by molecular heterogeneity with various immune cell infiltration patterns, which have been associated with therapeutic sensitivity and resistance. In particular, dendritic cells (DCs) are recently discovered to be associated with prognosis and survival in cancer. However, how DCs differ among ESCC patients has not been fully comprehended. Recently, the advance of single-cell RNA sequencing (scRNA-seq) enables us to profile the cell types, states, and lineages in the heterogeneous ESCC tissues. Here, we dissect the ESCC tumor microenvironment at high resolution by integrating 192,078 single cells from 60 patients, including 4379 DCs. We then used Scissor, a method that identifies cell subpopulations from single-cell data that are associated bulk samples with genomic and clinical information, to stratify DCs into Scissor^hi^ and Scissor^low^ subtypes. We applied the Scissor^hi^ gene signature to stratify ESCC scRNAseq patient, and we found that PD-L1, TIGIT, PVR and IL6 ligand-receptor-mediated cell interactions existed mainly in Scissor^hi^ patients. Finally, based on the Scissor results, we successfully developed a validated prognostic risk model for ESCC and further validated the reliability of the risk prediction model by recruiting 40 ESCC clinical patients. This information highlights the importance of these genes in assessing patient prognosis and may help in the development of targeted or personalized therapies for ESCC.

## Introduction

Esophageal cancer (EC) is one of the most prevalent cancers worldwide [1]. In China, esophageal squamous cell carcinoma (ESCC) is the most common histological subtype of EC. China alone accounts for 53% of ESCC cases in the world [2]. Previous studies have reported several risk factors for ESCC, including age, smoking, alcohol consumption and human papillomavirus [3]. However, the majority of ESCC patients are diagnosed at an advanced stage and the 5-year overall survival (OS) of ESCC remains unsatisfactory.

In recent years, studies have shed new light on immune cells in the tumor microenvironment (TME) as important prognostic and predictive biomarkers in ESCC [4]. Among different immune cell types, dendritic cells (DCs) are sentinel antigen-presenting cells (APCs) and exert an essential function in orchestrating immunity. Traditionally, DCs are characterized into tumor-infiltrating conventional DC type 1 (cDC1) and type 2 (cDC2), plasmacytoid DCs (pDCs) based on expression of certain subset-related markers or combination [5, 6]. However, these general markers were not able to fully discriminate between distinct human DC subsets. Over the past decade, the development of single-cell RNA sequencing (scRNA‐seq) has allowed the researcher to profile, identify, classify and discover new or rare DCs cell subtypes [7]. More importantly, Scissor [8], a recent developed algorithm, enables us to identify cell subpopulations from single-cell data which correlates with given phenotype based on clinical data collected from bulk RNAseq assays. Here, we aimed to establish and validate prognostic and diagnostic model for ESCC based on scRNA-seq and bulk-seq datasets. To do so, we screened a subpopulation of mature DCs closely related to poor OS of patients and obtained possible biomarkers, which could improve the prognosis of ESCC. This study improves the understanding of the heterogeneity and clinical relevance of DC subsets in ESCC.

## Materials and methods

### Data download

ESCC scRNA-seq data GSE160269 [9] including 60 ESCC patients, 17,986 genes and 192,078 cells was downloaded from GEO databases. TCGA clinical data and gene expression information including 79 ESCC patients were retrieved from the TCGA database (https://portal.gdc.cancer.gov/). ESCC bulk RNA-seq data GSE53625 [10] including 179 ESCC patients, 13,495 genes were download from GEO databases (https://www.ncbi.nlm.nih.gov/geo/).

### scRNA-seq data processing and clustering dimension reduction

To process the scRNA-seq data, we first used Seurat (V4.1.1) R package [11] to merge and normalized the data and identified the first 2000 highly variable genes via the FindVariableFeatures function “vst” method. We then used ScaleData function to scale all genes and performed RunPCA function to reduce the dimension of PCA for the first 2000 highly variable genes. We chose dim = 20 and clustered the cells via the “FindNeighbors” and “FindClusters” functions (resolution = 0.8) to identify the cell clusters. Subsequently, we chose the top 20 principal components to reduce dimensionality using the Uniform Manifold Approximation and Projection (UMAP) method. Lastly, we ran the FindAllMarkers function to select the marker genes of 38 clusters with logfc = 0.25 and Minpct = 0.25.

### Trajectory inference analysis

Trajectory analysis of DCs in ESCC was performed using CytoTRACE [12] and Monocle 2 [13]. CytoTRACE (V0.3.3) was performed based on the default recommended settings. When the calculation of the CytoTRACE algorithm is finished, each single cell will obtain a score that indicates its status of cell differentiation within the given dataset. For Monocle 2 (V2.24.0) analysis, we first obtained the DEGs between the clusters and applied them for dimension reduction through the reduceDimension function. Genes that changed along with the pseudotime were measured and visualized using the plot_pseudotime_heatmap function, and the genes were clustered into subgroups based on the gene expression patterns.

### Gene set variation analysis (GSVA)

Pathway analyses were performed on the 186 KEGG pathways retrieved from c2.cp.kegg.v7.2.symbols files. To assign pathway activity estimates to individual cells, we applied GSVA (V1.44.2) with standard settings. The significant pathways were selected according to the criterion: *p* < 0.05 and FDR < 0.25.

### SCISSOR analysis

SCISSOR (Version ‘2.0.0’) was used to associate phenotypic data from ESCC bulk RNA-seq data GSE53625 with ESCC scRNA-seq data GSE160269. SCISSOR was run on DCs of each patient individually according to the SCISSOR tutorial using overall survival (Cox regression) as dependent variables. A grid search for the alpha-parameter was performed, and a cutoff parameter of 0.00034 was used. Significant differences were identified by comparing the populations of Scissor^ +^ and Scissor^-^ cells using limma R package (Version ‘3.52.2’) and then screened with *p* < 1e10 and |log2FC|> 0.585 to identify the differences. In addition, KEGG functional enrichment analysis was performed using the Clusterprofiler (V4.4.4) package.

### Cell communication analysis and CIBERSORT estimation

For cell–cell communication analysis, CellChat R package was used (V1.5.0) with default parameters [14]. CIBERSORT [15] algorithm was designed to deconvolve tumor immune cell infiltration based on RNA-seq gene expression data. We calculated the relative proportion of 22 tumor-infiltrating immune cell subtypes in all TCGA-ESCC samples based on the CIBERSORT default settings.

### Construction of prognostic model

The DEGs associated with Scissor^ +^ DCs were selected as candidate genes for constructing a prognostic model. We then conducted least absolute shrinkage and selection operator (LASSO) Cox penalized regression analysis using the glmnet (V4.1–4) R package. Genes with nonzero coefficients were chosen to construct a risk score prognostic model. Based on the results of LASSO analysis, we calculated the risk score for each ESCC patient. Risk score = h0*e^∑_i_ = 0^n^exp(). Patients were grouped into high- and low-risk group based on the median-risk score. Kaplan–Meier (KM) curve combined with the log rank test was applied to compare the overall survival (OS) between two groups. Time-dependent receiver operating characteristic (ROC) was used to test the accuracy of prognostic model using timeROC R package. Next, uni-Cox and multi-Cox analyses were performed to examine the correlation between risk score, clinicopathological characteristics and OS of ESCC. We used ‘rms’ R package (V6.3.0) to generate a nomogram to predict 1-, 2- and 3-year OSs in TCGA_ESCC. Finally, we construct the calibration curve to evaluate the accuracy of nomogram-predicted OS.

### ConsensusClusterPlus

ConsensusClusterPlus is an R package that employs Consensus Clustering for analyzing high-dimensional data. This method is based on iteratively sampling data subsets from multiple random samples, which are subsequently clustered and integrated to yield more robust and stable clustering results.

### Survival analysis

Kaplan–Meier curves were primarily utilized for clinical prognostic analysis across various databases in this study. Figure 3E and F focuses on examining the clinical relevance of 58 upregulated genes (scissor signature). The GSVA score for each patient was calculated using both TCGA and GSE53625 datasets based on the ensemble of 58 genes, and the samples were divided into the “Scissor ^+^ ” group and the “Other” group using the median principle. In Figs. 6E, 7C and 8B, we calculated risk scores based on the coefficients using the following formula: risk score = (expression level of RPS24 * 0.348) + (expression level of MPP2 * 0.237) + (expression level of TRPM6 * − 0.256) + (expression level of SHISA9 * 0.136) + (expression level of CT83 * − 0.188) + (expression level of SPACA4 * − 0.117). We categorized the samples into high-risk and low-risk groups based on the median-risk score. P value was determined by the two-tailed log rank sum test.

### IHC staining

Tissue samples were fixed in 10% buffered formalin at room temperature overnight and then transferred to 70% ethanol for preservation and embedding. The prepared paraffin block cut into 5-μM slices was then deparaffinized, hydrated and sealed with 5% BSA for half an hour. After incubation overnight at 4 °C with the appropriate primary antibody at the optimal concentration, the sections were incubated with PBS and HRP secondary antibodies at the appropriate concentration for 30 min. Finally, the sections were stained with DAB staining solution from PBS and stained again with neutral background reagents. Microscopic observation and analysis of the sections is using image analysis software. The primary antibodies used in this study include TRPM6 (Proteintech, 55,455-1-AP, 1:200), MMP2 (Proteintech, 10,373-2-AP, 1:200), RPS24 (Proteintech, 14,831-1-AP, 1:200), CT83 (Proteintech, 25,708-1-AP, 1:200), SPACA4 (Novus, NBP2-38,913, 1:200), SHISA9 (Thermo Fisher, PA5-21,058, 1:200).

### Statistics

All statistical analyses were performed using the R software version 4.0.0. Univariate and multivariate Cox regression analyses were used to evaluate the prognostic value of factors. The Kaplan–Meier analysis with a 2-sided log rank test was used to compare the OS of patients. Statistical significance was set at *P* < 0.05 unless specified otherwise.

## Results

### Integration and clustering of ESCC scRNA-Seq data

**Fig. 1:**
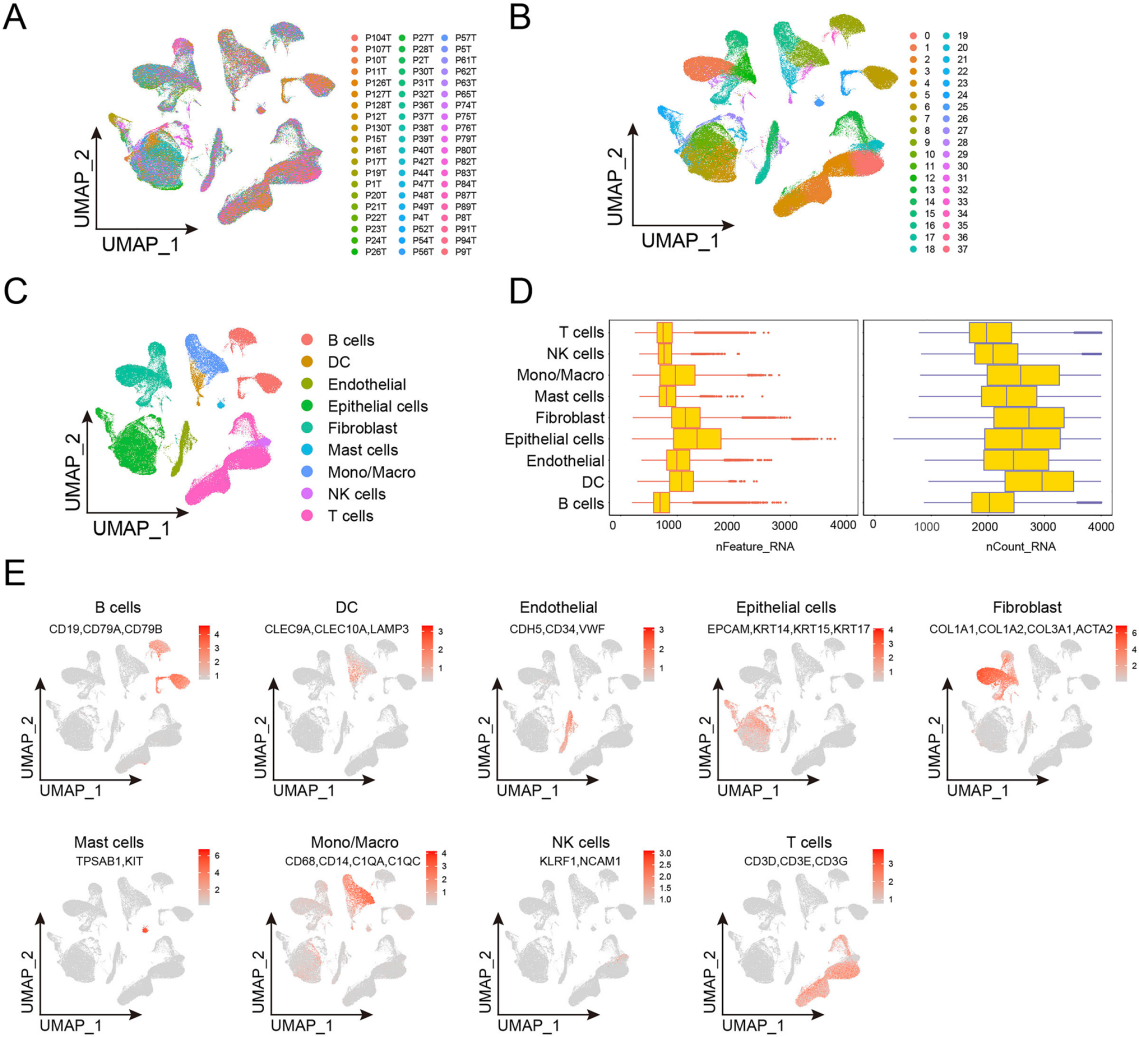
Dimensional reduction for scRNA-seq data from ESCC. **A**–**C** (**A**) UMAP of single-cell clusters from patients with esophageal cancer (*n* = 60), color based on various patients. (**B**) Assign colors to different Seurat clusters. (**C**) Give the various cell types distinct colors. **D** Box plots of the quantity of UMIs (left) and genes (right). **E** UMAP plots that display the expression of specific markers across all cell clusters

To investigate the cellular heterogeneity in ESCC, we first downloaded GSE160269 scRNA-seq dataset containing 60 ESCC patient samples and processed this dataset using Seurat package. Nonlinear dimensionality reduction was performed using UMAP approach (Fig. 1A). We used FindCluster function to cluster cells into in 38 clusters (Fig. 1B). Epithelial cells (clusters 5, 7, 10, 11, 21, 22, 23, 26 and 27; markers *EPCAM*, *KRT14*, *KRT15* and *KRT17*), fibroblasts (clusters 1, 12, 15, 18, 29, 32 and 34; markers *COL1A1*, *COL2A1*, *COL3A1* and *ACTA2*), endothelial cells (clusters 14, 17 and 28; markers *CDH5*, *CD34* and *VWF*), T cells (clusters 0, 2, 3, 4, 13, 35 and 37; markers *CD3D*, *CD3E* and *CD3G*), B cells (clusters 6, 8, 24, 33 and 36; markers *CD19*, *CD79A* and *CD79B*), monocytes and macrophages (clusters 9, 16 and 31; markers *CD68*, *CD14*, *C1QA* and *C1QC*), DC (clusters 20 and 30; markers *CLEC9A*, *CLEC10A*, and *LAMP3*), NK cells (clusters 19; markers *KLRF1* and *NCAM1*) and mast cells (clusters 25; markers *TPSAB1* and *KIT*) were classified according to cell markers (Fig. 1C and E). The average number of unique molecular identifiers (UMI) per cell was about 7,316, and a median of approximately 2,263 genes was detected per cell (Fig. 1D).

### Subtypes of DCs in the TME of ESCC

**Fig. 2:**
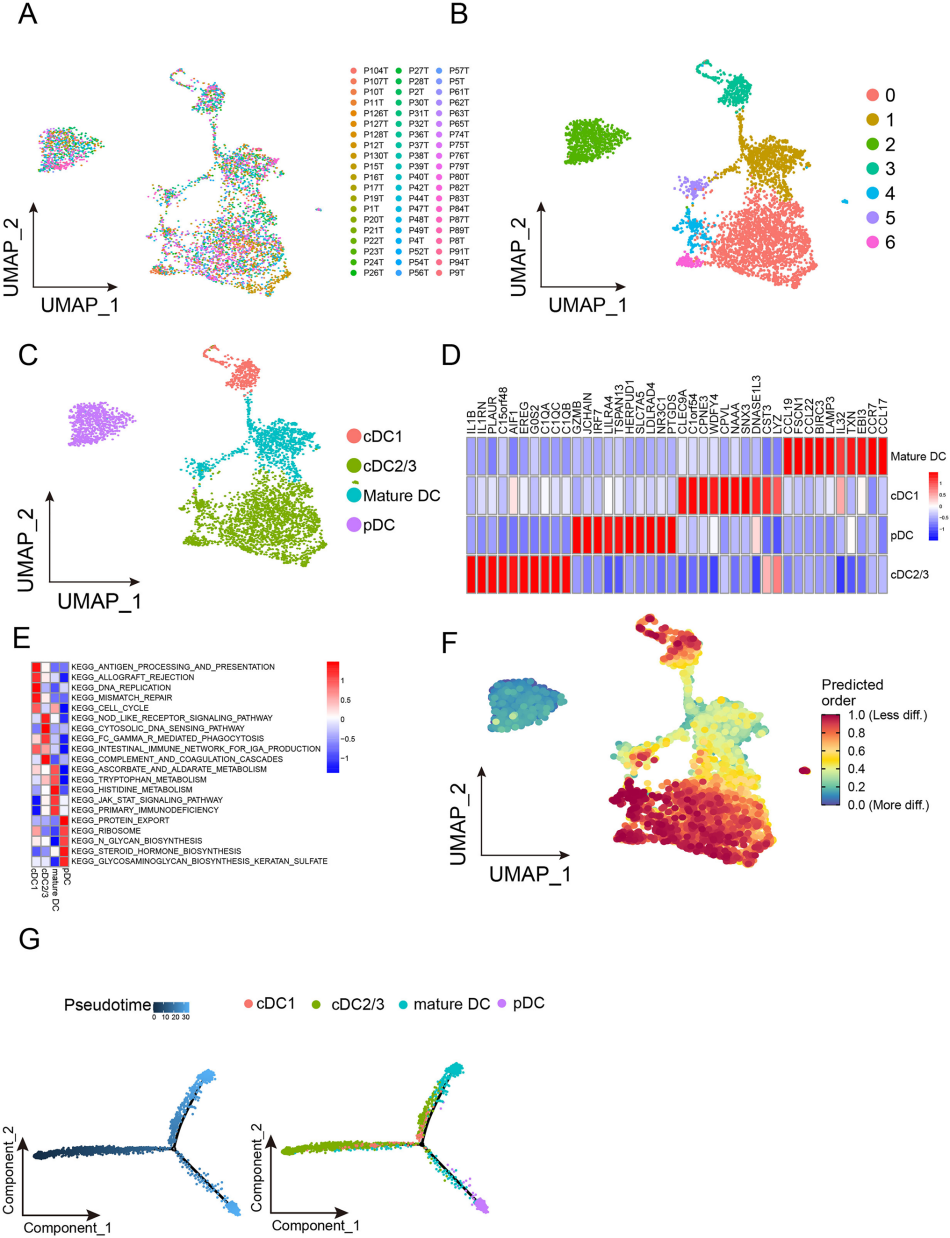
Identification of the dendritic cell population enriched in ESCC. **A**–**C** (**A**) UMAP plot of dendritic cells from 60 patients with esophageal cancer, color based on various patients. (**B**) Assign colors to different Seurat clusters. (**C**) Give the various cell types distinct colors. **D** Signature gene heatmap for four cell groupings of dendritic cells. Ten particularly expressed genes serve as the representation for each cell cluster. **E** Heatmap showing distinct pathways that were found to be abundant in the different cell type of dendritic cells using GSVA analysis. **F** UMAP plots displaying the dendritic cells’ distribution of CytoTRACE scores. Greater stemness is indicated by higher scores. **G** All dendritic cells in a monocle’s pseudotime trajectory of cell differentiation are shown on the left, along with four clusters of dendritic cells (right)

To dissect the heterogeneity and potential role of DCs, we extracted them for further investigation. Totally, we were able to obtain 4379 DCs from 60 ESCC samples (Fig. 2A). These DCs were divided into 7 subclusters (Fig. 2B). Among the 7 subclusters, cluster 3 was identified as cDC1 based on the presence of *CLEC9A* genes, clusters 0, 4 and 6 were identified as cDC2/3 (*C1QA*, *C1QB* and *C1QC*), cluster 2 was identified as pDC (*IRF7* and *SLC7A5*), and clusters 1 and 5 were identified as mature DC (*CCR7*, *LAMP3* and *CCL22*) (Fig. 2C and D). Our GSVA results show that the cDC1 was mainly enriched in the pathways and gene sets correlated with antigen processing and presentation, allograft rejection, DNA replication. The cDC2/3 was mainly enriched in pathways and genes relevant to Nod-like receptor signaling pathway, cytosolic DNA sensing pathway, FcγR-mediated phagocytosis. The mature DC was mainly enriched in pathways related to ascorbate and aldarate metabolism, tryptophan metabolism, histidine metabolism, JAK-STAT signaling pathway and primary immunodeficiency. The pDC were enriched in pathways and gene relevant to protein export, ribosome and N-glycan biosynthesis (Fig. 2E). We then used CytoTRACE to predict the differentiation state of DCs. We observed that the pDCs and mature DCs were more differentiated, while the cDC1 and cDC2/3 were less differentiated (Fig. 2F). To validate our findings, we also performed pseudotime analysis using the Monocle 2 software to determine a cell fate trajectory for DCs. Our trajectory analysis revealed a continuum of cells with three distinct branch points, showing a root corresponding predominantly to cDC2/3 and two terminal populations corresponding to pDCs and mature DC (Fig. 2G).

### Identifying DC subpopulation related to worse survival of ESCC

**Fig. 3:**
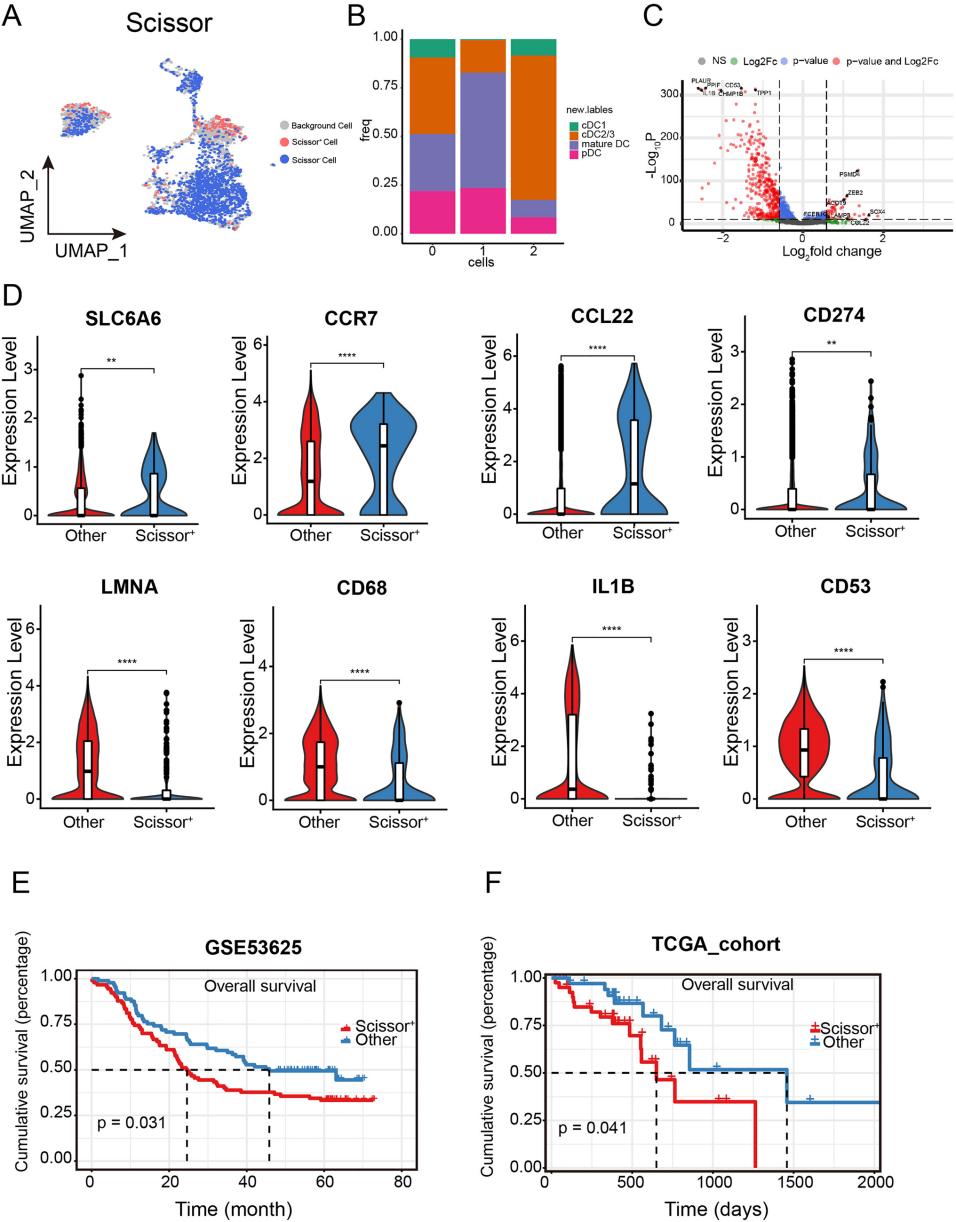
Identification of Scissor^+^ DCs of ESCC. **A** Scissor-selected cells’ UMAP plots. Scissor^+^ (poor survival) and Scissor^−^ (excellent survival) cells are represented by the red and blue dots, respectively. **B** The proportion of Scissor^+^ cells in various DC clusters is displayed using a bar plot (0 represents background cells, 1 represents Scissor^+^ cells, and 2 represents Scissor ^-^ cells). *P* values were presented by Chi-square test. *p* < 0.05. **C** Volcano plot of differential gene expressions in Scissor^+^ cells versus Scissor^−^ cells. The gene passed p value and fold change thresholds (*p* value < 0.05; fold change ≥ 2 or ≤ -2) was shown in red. **D** Violin plots of expression levels of differential genes in Scissor^ +^ cells and others. The FDR was the adjusted *P* value calculated by the t test. *P* values were presented by two-tailed unpaired Student’s t test. **E** The Kaplan–Meier survival curve illustrates the clinical relevance of the scissor signature, using GSE53625 datasets. Events that were censored are marked with a tick. *P* value was determined by the two-tailed log rank sum test. **F** The Kaplan–Meier survival curve illustrates the clinical relevance of the scissor signature, using TCGA-ESCC datasets. Events that were censored are marked with a tick. *P* value was determined by the two-tailed log rank sum test

To dissect which DCs are related to the poor prognosis of ESCC in the scRNAseq dataset, we performed Scissor analysis, guided by 179 ESCC bulk samples (GSE53625) with survival information. We identified 192 Scissor^+^ DCs that were associated with worse survival and 1,680 Scissor^−^ DCs that were associated with better survival (Fig. 3A). Notably, mature DCs accounted for the highest proportion among Scissor^+^ DCs (p < 0.05, Fig. 3B). To further investigate the characteristics of Scissor^+^ DCs, we compared the gene expressions of Scissor^+^ DCs with all other DCs. We found 58 upregulated genes and 417 downregulated genes were differentially expressed in Scissor^+^ DCs over all other DCs, respectively (Fig. 3C). Importantly, we found *SLC6A6*, *CCR7*, *CCL22* and *CD274* were among the upregulated genes (Fig. 3D). Meanwhile, *LMNA*, *CD68*, *IL1B* and *CD53* were downregulated in the Scissor^+^ DCs (Fig. 3D). To study the clinical relevance of the 58 upregulated genes, we used TCGA and GSE53625 datasets. Results showed that patients with higher signature scores of the 58 upregulated genes had substantially worse OS than those with lower signature scores (Fig. 3E and F).

### Characteristics of cell–cell communications in scissor high ESCC

**Fig. 4:**
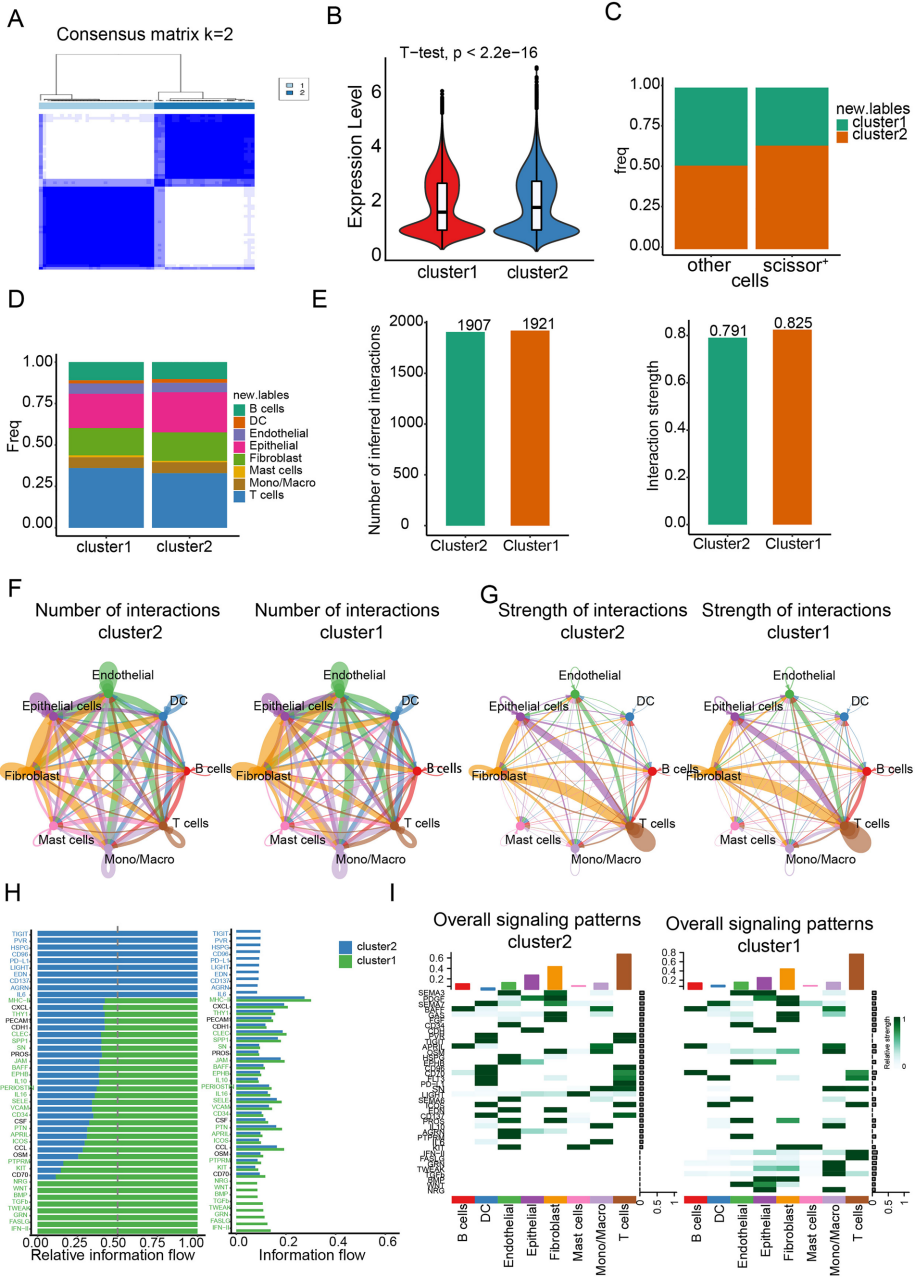
Molecular characteristics and cell–cell communications in scissor high ESCC patients. **A** Heatmap depiction of the consensus matrix showing NMF clustering results using 58 upregulated gene expression data from ESCC scRNA-seq dataset (k = 2). **B** Violin plot depicts signature scores of the 58 upregulated genes among two clusters of ESCC scRNA-seq data. *P* value was presented by two-tailed unpaired Student’s t test. **C** The proportion of Scissor^+^ cells among cluster1 and cluster2 of ESCC scRNA-seq data is displayed using a bar plot. **D** The proportion of different cell types among cluster1 and cluster2 of ESCC scRNA-seq data is displayed using a bar plot. **E** Cellchat showing the overall number and strength of intercellular communication. **F**–**G** Cellchat showing the overall number (F) and strength (G) of intercellular communication in cluster1 and cluster2, respectively. **H** Cellchat showing the difference in ligand–receptor pairs and molecular interactions among cell types in cluster1 and cluster2. **I** Cellchat showing the major signaling inputs and outputs among cluster1 and cluster2

To comprehensively profile the tumor microenvironment (TME) in esophageal squamous cell carcinoma (ESCC) samples with high expression of the scissor signature, we applied the 58 previously identified upregulated genes to ESCC single-cell RNA sequencing (scRNAseq) samples. Using the R ‘ConsensusClusterPlus’ package for unsupervised clustering, we aimed to uncover distinct subpopulations within the TME. Based on the cumulative distribution function (CDF), we chose k = 2 as the optimal clustering parameter to classify the ESCC samples into two clusters, and we identified two distinct clusters: Cluster 1 contained 32 cases, while Cluster 2 contained 28 cases (Fig. 4A). We found that cluster 2 displayed higher signature scores of the 58 upregulated genes and harbored a higher proportion of Scissor^+^ DCs (Fig. 4B and C). Comparing the cellular composition, we found the proportions of all cell types were distributed similarly between these two clusters (Fig. 4D). Cellchat was then used to profile the overall communication atlas between two clusters. In general, we found the number and strength among different cell types were comparable between cluster 1 and cluster 2 (Fig. 4E–G). Subsequently, we compared the difference in ligand–receptor pairs and molecular interactions among cell types in both clusters. The results showed that multiple ligand–receptor-mediated cell interactions existed mainly in cluster2, including PD-L1, TIGIT, PVR, HSPG and IL6 (Fig. 4H). By contrast, NRG, WNT, BMP, TGFb and IFN-II signaling pathways were mainly enriched in the cluster1 (Fig. 4H). In addition, some signaling pathways like MHC-II and ICOS are decreased in cluster2 (Fig. 4H). We found that PD-L1 and TIGIT could be highly secreted by DCs and T cells in cluster2, whereas IFN-II and FASLG are mainly expressed by T cells and monocytes/macrophages in cluster1 (Fig. 4I).

### Identification of scissor high samples in bulk RNA-Seq data

**Fig. 5:**
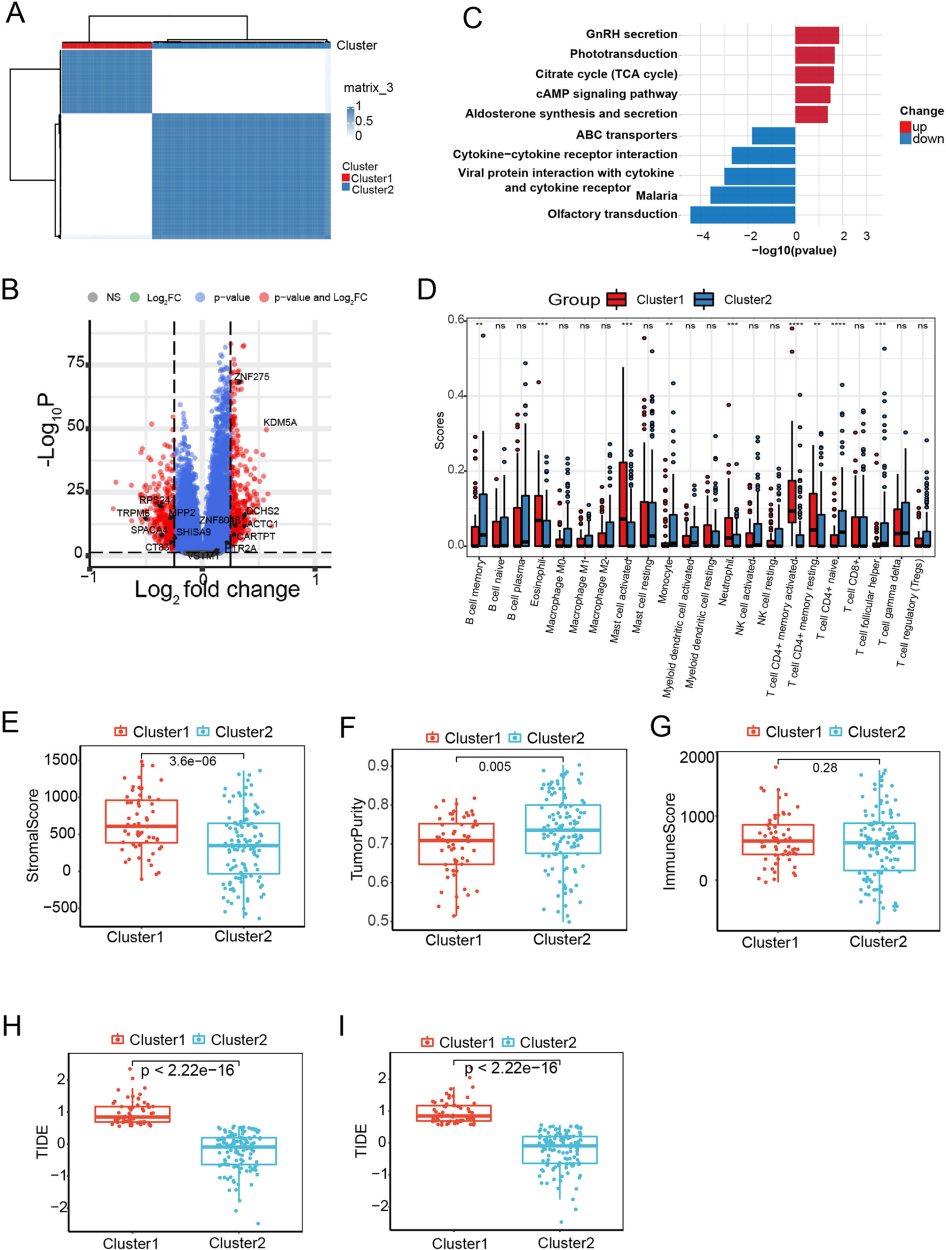
Identification of Scissor high samples in Bulk RNA-Seq Data. **A** Heatmap depiction of the consensus matrix showing NMF clustering results using 58 upregulated gene expression data from the GSE53625 dataset. (k = 2). **B** Volcano plot of differential gene expressions in cluster1 versus cluster2. **C** KEGG pathway analysis of the up- and downregulated differentially expressed genes among cluster1 and cluster2. **D** Box plot of 22 types of immune infiltrating cells in the cluste1 and cluster2. **E**–**G** Box plot depicts stromal score (E), Tumor purity score (F) and immune score (G) in cluster1 and cluster2. *P* values were presented by two-tailed unpaired Student’s t test. H and I Box plot of TIDE score between the cluster1 and cluster2. *P* values were presented by two-tailed unpaired Student’s t test

To study the role of these 58 genes further, we used them to cluster 179 ESCC sample in the GSE53625 dataset. We performed consensus clustering of the 58 up-regulated genes, and based on cumulative distribution function (CDF) analysis, we determined that choosing k = 2 as the optimal clustering parameter yielded consistent results. Therefore, we divided the 179 esophageal squamous cell carcinoma (ESCC) samples into two distinct clusters (Fig. 5A). We then compared the transcriptomes of these two clusters and performed pathway enrichment analysis. We identified 207 upregulated genes and 217 downregulated genes in cluster1 bulk samples and found cluster1 ESCC expressed higher level of Scissor^+^ genes (Fig. 5B). KEGG analysis revealed that Cluster1 ESCC samples upregulated pathways controlling GnRH secretion, phototransduction, TCA cycle, cAMP signaling pathway and aldosterone synthesis and secretions, while cluster2 ESCC samples were enriched in pathways related to olfactory transduction, cytokine–cytokine receptor interaction and ABC transporters (Fig. 5C). To evaluate the immune cell infiltration in both clusters, we used CIBERSORT to analyze the proportion of immune cells using bulk RNA-seq data. We found eosinophil, activated mast cells, neutrophil and activated memory CD4^+^ T cells and resting memory CD4^+^ T cells were highly expressed in cluster1 samples. On the contrary, memory B cells, monocytes, naïve CD4^+^ T cells and follicular helper T cells were upregulated in cluster2 ESCC samples (Fig. 5D). To probe the relative contributions of stromal and immune cells in these ESCC clusters, we utilized the ESTIMATE algorithm and found that cluster1 ESCC patients exhibited a significantly higher degree of stromal cells relative to cluster2 ESCC patients, with stromal scores and tumor purity scores differing significantly between these groups even though immune scores did not (Fig. 5E–G). Finally, we obtained TIDE prediction score of each ESCC patient. A higher TIDE prediction score suggests a higher potential for immune evasion and less likely to benefit from immune checkpoint inhibitor (ICI) therapy. Importantly, we found the TIDE scores of the cluster1 patients were significantly higher than those of the cluster2 patients (Fig. 5H and I).

### Establish an effective prognostic risk model for ESCC

**Fig. 6:**
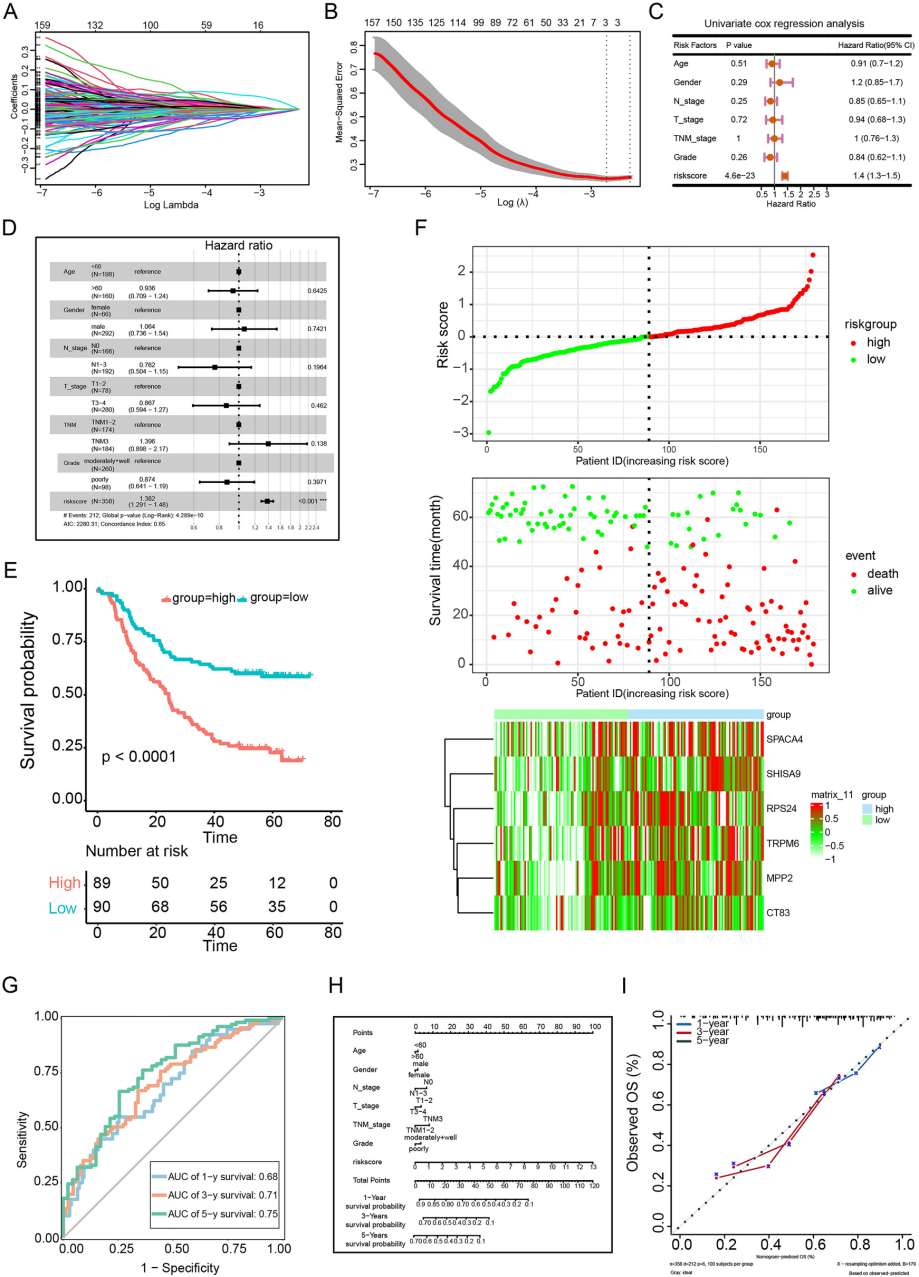
Construction of a prognostic risk model for ESCC from GEO datasets. **A** LASSO regression was used for variable screening. **B** LASSO coefficient profiles, a coefficient profile plot was produced against the log(λ) sequence. **C** Single-factor Cox regression analysis of OS-related clinical factors. **D** A multiple regression logistic analysis of parameters that were associated with survival. E Kaplan–Meier curve between high- and low-risk groups.* P* value was determined by the two-tailed log rank sum test. **F** The model divides the training set patients into low-risk or high-risk groups. **G** ROC curve graphs for overall survival at 1, 3 and 5 years. **H** and **I** The nomogram model and calibration curve used to predict the OS of patients with ESCC at 1, 3 and 5 years

**Fig. 7:**
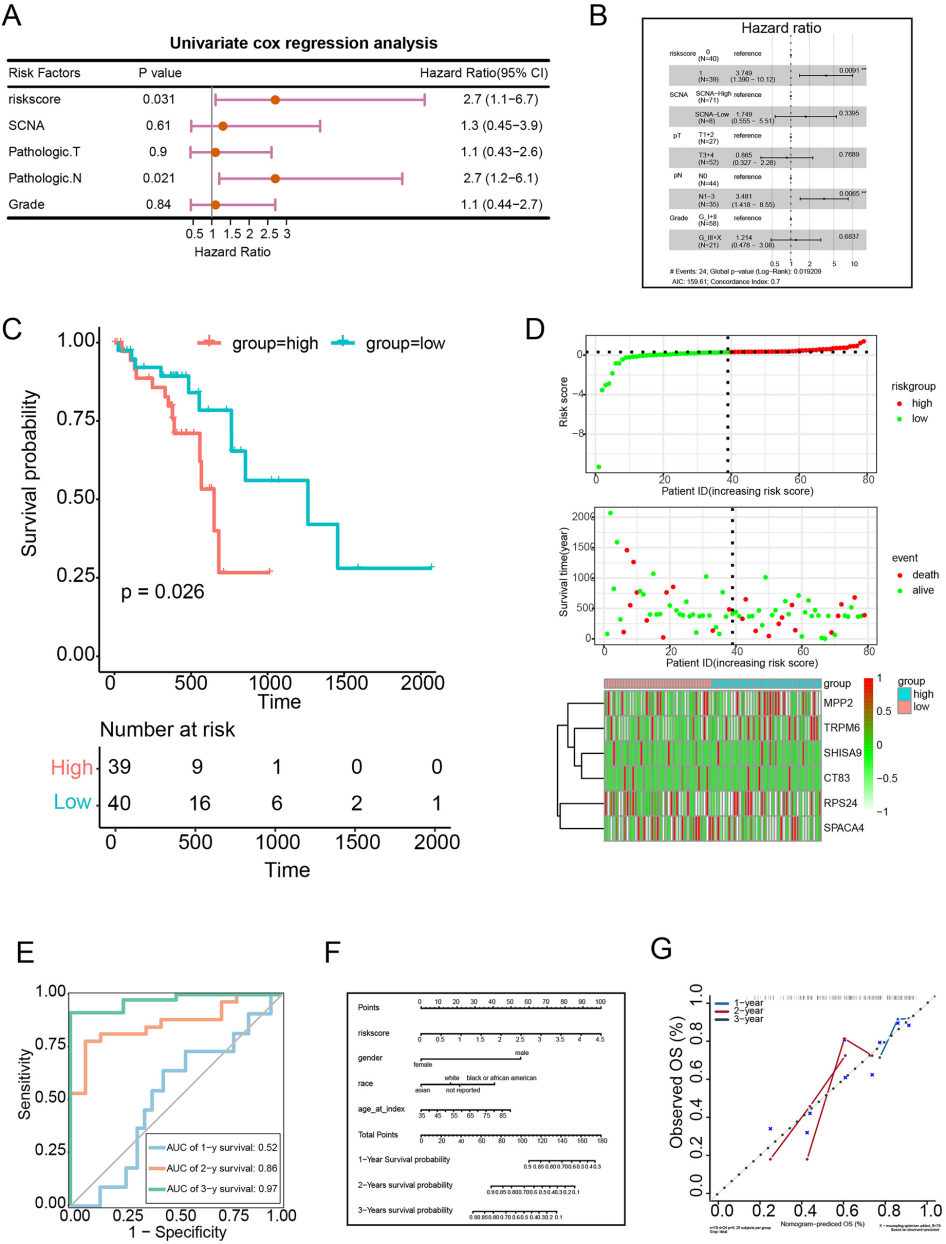
Validation of the prognostic risk model for ESCC using TCGA datasets. **A** and **B** Single-factor(A) and multi-factors(B) Cox regression analysis of OS-related clinical factors. **C** The model divides the training set patients into low-risk or high-risk groups.* P* value was determined by the two-tailed log rank sum test. **D** Single-factor Cox regression analysis of OS-related clinical factors. **E** ROC curve graphs for overall survival at 1, 2 and 3 years. **F** and **G** The nomogram model (F) and calibration curve (G) used to predict the OS of patients with ESCC at 1, 2 and 3 years

To construct a prognostic risk model, we performed LASSO regression analysis to reduce the number of DEGs, with six genes identified, including RPS24, MPP2, TRPM6, SHISA9, CT83 and SPACA4 (Fig. 6A and B). According to their coefficients, we calculated the risk score according to the following formula: risk score = expression level of RPS24* 0.348 + expression level of MPP2* 0.237 + expression level of TRPM6* (− 0.256) + expression level of SHISA9* (0.136) + expression level of CT83 *(− 0.188) + expression level of SPACA4 * (− 0.117). To test whether the risk score was able to predict prognosis independently of traditional clinical features, we performed univariate and multivariate Cox regression analyses in both GSE53625 cohort and TCGA cohort. We found risk score (HR = 1.4 and 1.382, 95% confidence interval (CI): 1.3–1.5 and 1.291–1.48, respectively) was independent predictor of OS (Fig. 6C and D). Analysis of the TCGA-ESCC cohort confirmed that the risk score (1.1 and 3.749, 95% CI: 0.44–2.7 and 1.39–10.12, respectively) was independent predictor of OS (Fig. 7A and B). We then divided patients into high- and low-risk groups according to the median value of risk score in both GSE53625 cohort and TCGA cohort. KM and log rank analysis showed that ESCC patients in the high-risk group were associated with the worse OS when compared with ESCC patients in the low-risk group in both datasets (Fig. 6E and Fig. 7C). Figures 6F and 7D indicate the total risk score (upper panel), survival time (middle panel) and gene expression levels (lower panel) for the GSE53625 and TCGA-ESCC datasets. Moreover, the risk score performed well in predicting the OS in the GSE53625 cohort (AUC for 1-, 3- and 5- year OS: 0.68, 0.71 and 0.75; Fig. 6G) and TCGA-ESCC cohort (AUC for 1-, 2- and 3- year OS: 0.52, 0.86 and 0.97; Fig. 7E). We also generated nomograms incorporating different factors to predict the 1-, 3- and 5-year OS in the GSE53625 dataset (Fig. 6H) and the 1-, 2- and 3-year OS in the TCGA-ESCC dataset (Fig. 7F). The calibration curves suggested that the nomograms were well-calibrated across the datasets (Figs. 6I and 7G).

### Clinical validation of risk modeling

**Fig. 8:**
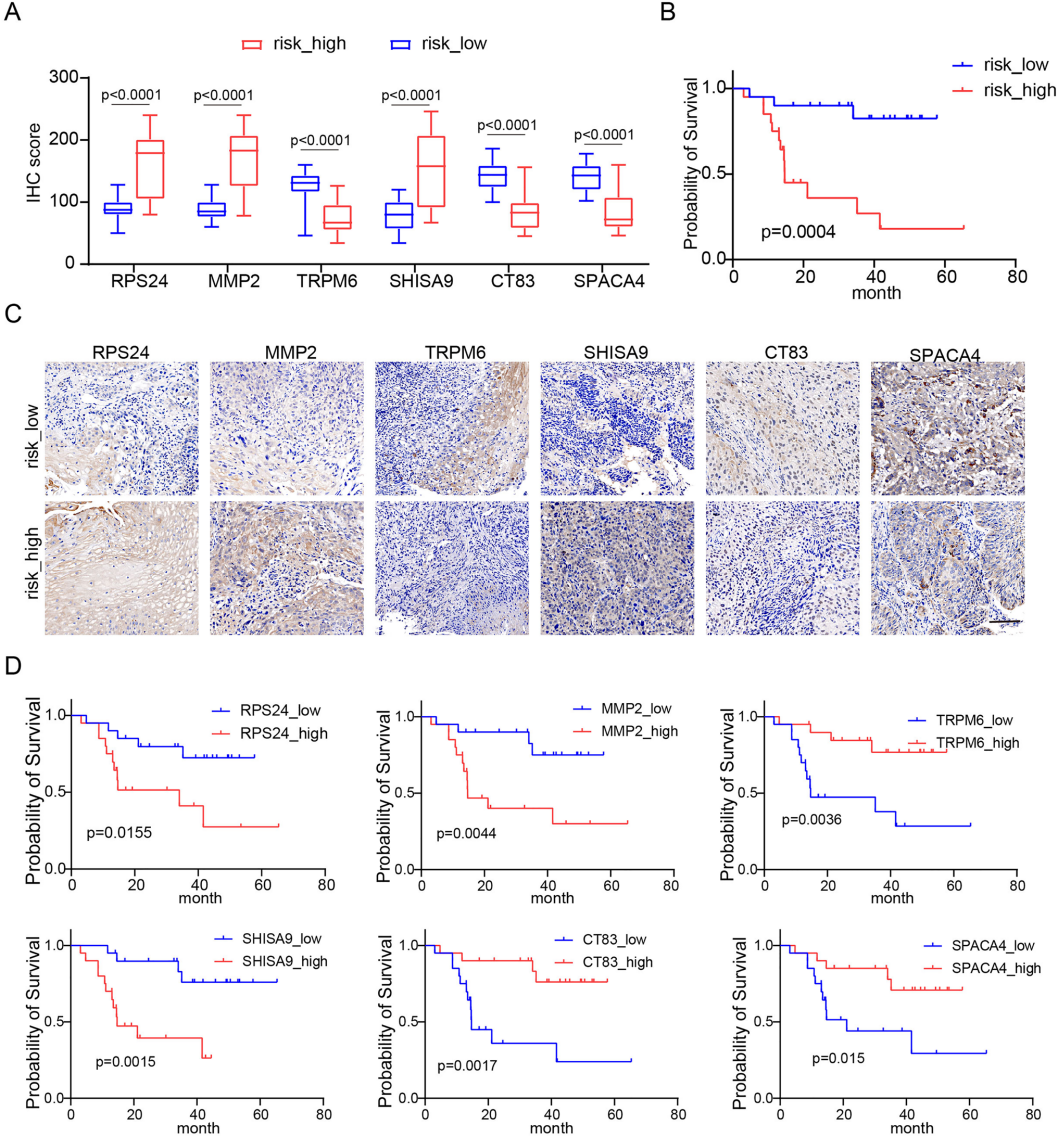
Validating the risk model genes in a clinical group. **A** Expression levels of the six genes were compared between the high-risk and low-risk groups.* P* values were presented by two-tailed unpaired Student’s t test. **B** Kaplan–Meier survival curves of ESCC patients based on risk scores.* P* value was determined by the two-tailed log rank sum test. **C** The representative immunohistochemical image is derived from the six genes in the risk model that are in the high-risk and low-risk groups. Scale bar, 100 μm. **D** Kaplan–Meier survival curves of ESCC patients based on six genes in the risk model

To confirm the prognostic significance of our model, we collected a clinical cohort of 40 patients diagnosed with esophageal squamous cell carcinoma (ESCC) at various clinical stages from the First Hospital of Sun Yat-Sen University. The primary objective was to validate the expression of six specific genes (RPS24, MPP2, TRPM6, SHISA9, CT83 and SPACA4) by directly measuring their protein expression levels by immunohistochemistry. Our goal was to strengthen the association between these genes and our risk model and to accurately discriminate low-risk and high-risk patients in clinical cohorts based on immunohistochemistry scores combined with risk model correlation coefficients. The risk score of the patient was calculated by using the immunohistochemical scores of the six key genes and their respective risk coefficients. Based on the median-risk score, patients were then divided into high-risk and low-risk groups. Our results showed significant differences in the expression of these genes between high-risk and low-risk populations. RPS24, MPP2 and SHISA9 were significantly overexpressed in the high-risk group. TRPM6, CT83 and SPACA4 were significantly underexpressed in the high-risk group (Fig. 8A, C). Furthermore, we performed Kaplan–Meier survival analysis and observed a significant correlation between ESCC patients in the high-risk group and poor prognosis (Fig. 8B). Further analysis revealed that ESCC patients with high expression of RPS24, MPP2 and SHISA9 had a poor prognosis, indicating that these genes functioned as risk factors. Conversely, ESCC patients with high expression of TRPM6, CT83 and SPACA4 had a better prognosis, suggesting that these genes function as protective factors (Fig. 8D).

These experimental results are consistent with the risk model we developed and support the conclusion that these six genes hold promise as potential prognostic biomarkers for predicting the survival outcomes of ESCC patients. This information underscores the importance of these genes in assessing patient prognosis and may aid in the development of targeted therapies or personalized treatment approaches for ESCC.

## Discussion

ESCC is well-recognized by their intra-tumor and inter-tumor heterogeneities, which brings major challenge for effective treatment of ESCC. Besides malignant cells, various types of cells in TME also contribute greatly in tumor heterogeneity. Previously studies have shown that the abundance of T cells infiltration correlates with the level of malignancy and patient prognosis [16]. Meanwhile, other cell types in TME, such as macrophages and neutrophils, are also involved in the regulation of tumor immunity [17, 18]. Hence, the response of different patients to immunotherapy has an extensive heterogeneity. Furthermore, there exist complicated cell–cell interactions between different cell types in the TME, increasing the complexity of tumor development. It is therefore important to dissect these cell–cell communications in order to develop effective anticancer immunotherapy strategies.

In the process of oncogenesis, the different DC subtypes are localized in and/or recruited to tumors. However, different DC subtypes display distinct functions in the tumor setting. For example, pDC can act to elicit enhanced anti-tumor immunity via production of type I interferons (IFN-I) [19]. In addition, cDC1 and cDC2 are effective antigen-presenting cells which can induce T cell-mediated immune responses against tumor [20]. On the contrary, DCs can also be guided to suppressive mechanisms and promote tumor development. The phagocytic ability of DCs decreases along maturation process. However, mature DCs can upregulated various molecules, including CD80, ICOSL, PD-L1, PD-L2 and CCR7 [21, 22]. The expression of PD-L1 in mature DCs is induced by receptor tyrosine kinase AXL [22]. Both cDC1 and cDC2 cells are programmed to differentiate into this regulatory subset upon uptake of tumor antigens [23]. Consistently, here we identified Scissor^+^ DCs are mainly mature DCs derived from both cDC1 and cDC2 subpopulations, suggesting mature DCs might be responsible for worse outcome of ESCC patients. However, in ovarian cancer, mature DCs correlate with favorable immune infiltration and improved prognosis of patients [24], suggesting a context-dependent role of mature DCs in tumors.

Specifically, we obtained 58 upregulated genes in Scissor^+^ DCs from scRNA-seq data from 60 ESCC patients. In addition, the Kaplan–Meier analysis confirmed a significantly shortened OS for ESCC patients with a high score of these 58 upregulated genes in both GEO and TCGA-ESCC datasets. Importantly, results from our TIDE analysis suggested that patients with higher Scissor^+^ DCs score are less likely to respond to ICI therapy. We also constructed a ESCC prognosis model using LASSO regression analyses to construct a signature of six genes. In addition, the univariate and multivariate Cox regression analyses showed that the risk score may be an independent predictor of OS of ESCC. Finally, our nomogram showed outstanding prediction in both GEO and TCGA-ESCC datasets, indicating that it may be able to predict ESCC patient survival in the clinical setting.

## Data Availability

The ESCC scRNA-seq datasets accession number GSE160269 is analyzed during the current study that is available in the GEO database repository. The ESCC bulk RNA-seq datasets accession number is GSE53625 that was downloaded from GEO databases (https://www.ncbi.nlm.nih.gov/geo/).
